# Intramyocardial Bridging: An Overlooked Cause of Atypical Chest Pain

**DOI:** 10.7759/cureus.60874

**Published:** 2024-05-22

**Authors:** Mohamed R Abouzid, Ibrahim Kamel, Sadaf Esteghamati, Kareem Mohamed, Amr Hamed

**Affiliations:** 1 Internal Medicine, Baptist Hospitals of Southeast Texas, Beaumont, USA; 2 Internal Medicine, Tufts Medical Center, Boston, USA; 3 Internal Medicine, Steward Carney Hospital, Boston, USA; 4 Internal Medicine, University of La Verne, La Verne, USA; 5 Internal Medicine, University of Missouri-Kansas City, Kansas City, USA

**Keywords:** myocardial ischemia, intramyocardial bridging, revascularization, atherosclerosis, coronary angiography, sudden cardiac death, myocardial infarction

## Abstract

Intramyocardial bridging (IMB) is a congenital anomaly characterized by the tunneling of a coronary artery segment through the myocardium, potentially leading to serious cardiac complications, such as myocardial ischemia, infarction, and sudden death, challenging the traditional view of it being benign. A case involving a 42-year-old man with a seven-day history of atypical chest pain highlights the significance of considering IMB in the differential diagnosis. Despite normal troponin levels, creatine kinase (CK), CK-MB, D-dimer, a negative drug screen, a normal ECG, and chest X-ray and no apparent issues on echocardiogram, left heart catheterization revealed IMB in the left anterior descending artery. This case underscores the necessity of including IMB in the differential diagnosis for chest pain, particularly in young males with familial cardiovascular disease history. While noninvasive imaging methods are useful for diagnosis, coronary angiography is the definitive diagnostic tool. Treatment primarily involves beta-blockers and calcium-channel blockers, with revascularization as a secondary option for those unresponsive to medication.

## Introduction

Intramyocardial bridging (IMB) impacts the coronary arteries, which commonly traverse the space between the pericardium and epicardium. In IMB, a segment of the coronary artery travels through the myocardium, covered by muscle fibers, which are referred to as a "myocardial bridge," causing systolic and diastolic flow disturbance. This intramyocardial segment is known as a "tunneled artery." While IMB was traditionally considered a benign anomaly, recent studies have suggested that it may cause significant cardiac effects in some patients, such as myocardial ischemia, myocardial infarction, and sudden cardiac death [1,2].

The precise prevalence of IMB remains uncertain; nevertheless, it is expected to be present to varying extents in around one-third of the adult population [3]. Coronary angiography (CA), coronary computed tomography angiography (CCTA), and autopsy examinations are the main methods used to determine the prevalence of IMB in the general population. Autopsy investigations are widely regarded as the most reliable method for determining IMB and have been established as the gold standard in this regard. These studies have reported a prevalence rate of IMB ranging from approximately 33% to 42% in the population. The disparity in prevalence observed among imaging modalities in different research studies is mostly influenced by the heterogeneity of the target groups, the specific imaging devices employed, and the decision to include or exclude "superficial" IMB. The left anterior descending artery (LAD) is the coronary artery most frequently impacted in terms of anatomical location. Approximately 67-98% of IMBs are found in this region, predominantly in the proximal and mid-LAD segments. Less frequently, the left circumflex and right coronary arteries are impacted [4].

While most cases of IMB are benign, a subset of symptomatic patients may present with significant cardiac effects, which can represent a diagnostic and therapeutic challenge. The emergence of modern functional and anatomical imaging techniques, including both invasive and noninvasive methods, has significantly enhanced our comprehension of the dynamic pathophysiology linked to IMB [2]. Hence, it is imperative for physicians to possess a thorough understanding of the epidemiology, pathobiology, diagnosis, functional assessment, and therapy of IMB in order to make well-informed decisions regarding patient care. Beta-blockers and calcium-channel blockers are considered first-line treatments, with revascularization reserved for those who do not improve with medical management alone. Revascularization techniques include percutaneous coronary intervention and surgical myotomy based on lesion length, depth, concurrent atherosclerosis, operator expertise, and patient preference [5]. 

The scholarly article authored by Sternheim et al. (2021) offers a comprehensive overview of the current understanding of IMB, encompassing its diagnostic procedures, functional evaluation, and treatment strategies. The authors highlight the potential hemodynamic significance of MB, citing several studies that suggest a correlation between MB and various cardiovascular pathologies, including acute myocardial infarction, ventricular rupture, life-threatening arrhythmias, hypertrophic cardiomyopathy, apical ballooning syndrome, and sudden death [5].

## Case presentation

**Figure 1:**
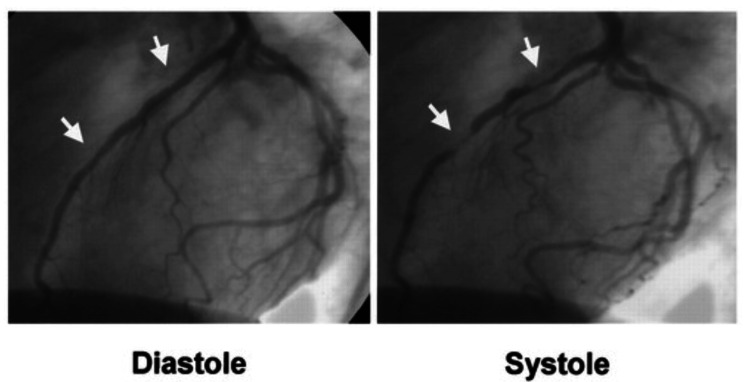
Coronary angiography findings

A 42-year-old man presented to the emergency room with seven days of chest pain. He characterized the pain as pressure-like, mild in intensity, intermittent, non-radiating, worsening at night and with deep breathing, and having no alleviating factors. His medical history indicated hyperlipidemia. He denied smoking or drinking alcohol. His family history was significant for heart attacks in his brother at the age of 46 and in his grandfather. Upon admission, three sets of troponins were negative (<0.012), and CK and CK-MB were within normal limits. D-dimer was negative. A urine drug screen was negative, minimizing the suspicion of drug-induced ischemia. The electrocardiogram (ECG) showed normal sinus rhythm with no ST-T changes. The chest X-ray showed clear lungs without acute abnormalities. The transthoracic echocardiogram showed no regional wall motion abnormalities or valvular disease, with an ejection fraction of 60-65%. Left heart catheterization was performed, which revealed a segment of IMB in the mid-left anterior descending artery after the take-off of the diagonal branch (see Figure 1). The patient was started on metoprolol, aspirin, and atorvastatin and discharged home. He was advised regular follow-ups with a cardiologist as an outpatient.

## Discussion

This case report underscores the need to comprehend the intricacy of IMB, a condition that was previously deemed clinically inconsequential. Although diastole accounts for approximately 85% of coronary blood flow, IMB is distinguished by systolic arterial compression, which poses a risk to only about 15% of coronary blood flow [5]. However, the complex interplay between anatomical and physiological factors that reciprocally influence each other in a dynamic manner during the cardiac cycle and throughout the patient's lifespan is quite intricate. The dimensions of the tunneled segment are crucial factors in determining the substrate that ultimately gives rise to ischemia symptoms in certain instances [6]. The effect of IMB is dynamic and exhibits variability in response to changes in the cardiac cycle, heart rate, and sympathetic tone. The sub-endocardium, which is more susceptible to ischemia, experiences the most dramatic delay in rapid early-diastolic hyperemia [7].

Increased sympathetic activity results in a higher heart rate and a shorter period of diastolic perfusion, hence worsening the loss of blood flow. The delay in rapid early-diastolic coronary flow caused by IMB significantly reduces the natural stress hyperemia response, therefore worsening the mismatch between supply and demand. In order to achieve a precise clinical evaluation of IMB, it is imperative to employ a method that encompasses the context of heightened sympathetic tone [8]. One significant pathophysiological consequence of ischemic IMB is the occurrence of "branch steal," particularly observed in septal perforator arteries [9]. In the end phase of systole and the initial phase of diastole, the blood flows via a constricted segment within the tunneled artery. The constriction of the septal branch leads to an increase in fluid velocity, which subsequently produces a decrease in perfusion pressure at the ostium. The Venturi effect is responsible for this phenomenon. Studies have shown evidence for the occurrence of "branch steal" in IMB, indicating that the diastolic pressures in the intrabridge segment are lower than those in the distal artery. In addition, it has been observed that mild-moderate IMBs frequently lead to local or septal ischemia, rather than ischemia in the distal myocardium [7].

The development of atherosclerotic coronary disease has been associated with IMB. Coronary arteries experience various mechanical forces, such as compressive stress, tensile stress, and shear stress, under typical circumstances [5]. Shear stress, also known as wall shear stress (WSS), refers to the tangential frictional force exerted on a flowing fluid. Extensive research has demonstrated its involvement in the pathogenesis of endothelial dysfunction and atherosclerosis, potentially through the induction of inflammatory pathway modifications [10]. Doppler investigations of IMBs demonstrate that when the tunneled artery is compressed during systole, it generates a flow that moves backward towards the IMB. This creates a region with low wall WSS, which has been suggested as a possible explanation for the formation of atherosclerotic plaque near the IMB. However, it is important to note that these altered biomechanical stresses could potentially contribute to certain nonatherosclerotic complications associated with IMB. The anticipated consequence of subjecting the tunneled artery to substantial compressive stress by the IMB is the occurrence of intimal injury, which possesses the capacity to advance into dissection [11,12]. In summary, IMB, a condition that was previously deemed clinically insignificant, is a multifaceted anatomical abnormality that necessitates precise clinical evaluation employing a technique that encompasses the context of heightened sympathetic tone. Anatomical and physiological elements interact in a dynamic manner throughout the cardiac cycle and the patient's lifespan. The delay in rapid early-diastolic coronary flow caused by IMB significantly reduces the normal stress hyperemia response, hence worsening the mismatch between supply and demand. IMB has been linked to the occurrence of atherosclerotic coronary disease, potentially caused by changes in biomechanical forces that lead to endothelial dysfunction and atherosclerosis. 

Understanding these factors can help improve the diagnosis and management of IMB [5]. Infrequently, these patients' resting electrocardiograms reveal abnormalities; consequently, other modalities are required to identify myocardial bridging [13]. CA continues to be the standard diagnostic method for identifying IMB. In the context of CA, the systolic compression of the tunneled artery is visually represented as a "milking effect," which is distinguished by the occurrence of retrograde blood flow during diastole, followed by antegrade flow. Additional diagnostic procedures that are considered invasive include intravascular ultrasonography and intracoronary Doppler sonography. The characterization of myocardial bridge length, thickness, and position is achieved through the utilization of intravascular ultrasonography. The presence of an echo-lucent zone between the bridging artery and the epicardial tissue, which is present throughout the cardiac cycle, is represented as a "half-moon" sign. Intracoronary Doppler imaging reveals a distinctive diastolic fingertip phenomenon associated with myocardial bridging. This phenomenon is distinguished by a fast diastolic blood flow, which is subsequently followed by a plateau in the segment that is bridged [14].

Noninvasive diagnostic techniques, such as cardiac computed tomography angiography (CCTA), cardiac magnetic resonance imaging (MRI), and transthoracic echocardiography, can also be employed for the identification of indwelling myocardial infarction (IMB); nevertheless, these are not the recommended choices for diagnosis. CCTA is a nascent diagnostic method for identifying coronary abnormalities. This diagnostic tool offers insights into the structure and composition of the coronary arteries, enabling the assessment of various conditions, such as coronary abnormalities, atherosclerosis, stent patency, bypass grafts, and cardiac irregularities. One advantage of this approach is its noninvasive nature, utilization of lower radiation doses, and its utility in treatment planning. While not considered the definitive diagnostic method, CCTA would have been a suitable option for diagnosing myocardial bridging in our patient. This is because the likelihood of detecting significant coronary atherosclerosis on angiography as the underlying cause of her condition is low due to her young age and low-risk factors [15]. Due to the absence of substantial guidelines from the cardiology society regarding the diagnosis or treatment of IMB, the management of symptomatic IMB continues to pose a therapeutic difficulty.

In this particular setting, it is imperative to take into account the symptoms exhibited by the individual patient, as well as their coronary and cardiac anatomy, degree of ischemia, and concomitant diseases. These factors have the potential to exert a substantial influence on the outcomes of patients with IMB [5]. Pharmacologic therapy is the primary treatment for the majority of people with symptomatic IMB. Beta-blockers are commonly considered the primary pharmacological treatment due to their adverse chronotropic and inotropic effects [16,17]. Furthermore, apart from the beneficial decrease in sympathetic drive, these interventions result in a drop in heart rate and an increase in diastolic filling time, hence facilitating the decompression of the tunneled section.

Calcium-channel blockers are commonly employed and favored in patients who have contraindications to beta-blockers [11]. Ivabradine, a targeted inhibitor of hyperpolarization-activated cyclic nucleotide-gated channels (f-channels) in the sinoatrial node, has the potential to serve as a secondary therapeutic option owing to its ability to decrease heart rate. The therapy may be considered for patients who exhibit intolerance to beta-blockers or calcium channel blockers or who fail to achieve a sufficiently regulated heart rate after receiving maximally tolerated treatment with these medications [18]. The emphasis should be placed on close clinical monitoring and modification of risk factors. If symptoms persist despite maximal medical therapy, revascularization should be considered using percutaneous coronary intervention (PCI) or surgery, such as coronary artery bypass grafting (CABG) or myotomy. The revascularization strategy must be guided by pre-procedural anatomic planning with CCTA [5,19].

Moreover, it is important to consider other potential differentials, such as gastroesophageal reflux disease, costochondritis, panic disorder, pulmonary conditions, musculoskeletal pain, pericarditis, aortic dissection, esophageal disorders, and various cardiac conditions. The thorough diagnostic process, including laboratory tests, imaging studies, and clinical evaluation, was instrumental in narrowing down the differential diagnoses and ultimately led to the diagnosis of IMB.

## Conclusions

This case report highlights the importance of considering IMB in the differential diagnosis of chest pain, particularly in young males with a positive family history of cardiovascular diseases. Non-invasive imaging techniques like echocardiography, cardiac CT, and cardiac MRI can be used for diagnosis, but invasive CA remains the gold standard. Medical therapy and close follow-up with a cardiologist are recommended for the management of IMB. Clinicians should be aware of the potential risks of myocardial ischemia and sudden death associated with IMB, and appropriate management should be implemented promptly.

## References

[1] Tarantini G, Migliore F, Cademartiri F, Fraccaro C, Iliceto S (2016). Left anterior descending artery myocardial bridging: a clinical approach. J Am Coll Cardiol.

[2] Hostiuc S, Rusu MC, Hostiuc M, Negoi RI, Negoi I (2017). Cardiovascular consequences of myocardial bridging: a meta-analysis and meta-regression. Sci Rep.

[3] Hostiuc S, Negoi I, Rusu MC, Hostiuc M (2018). Myocardial bridging: a meta-analysis of prevalence. J Forensic Sci.

[4] Rajendran R, Hegde M (2019). The prevalence of myocardial bridging on multidetector computed tomography and its relation to coronary plaques. Pol J Radiol.

[5] Sternheim D, Power DA, Samtani R, Kini A, Fuster V, Sharma S (2021). Myocardial bridging: diagnosis, functional assessment, and management: JACC state-of-the-art review. J Am Coll Cardiol.

[6] Uusitalo V, Saraste A, Pietilä M, Kajander S, Bax JJ, Knuuti J (2015). The functional effects of intramural course of coronary arteries and its relation to coronary atherosclerosis. JACC Cardiovasc Imaging.

[7] Klues HG, Schwarz ER, vom Dahl J (1997). Disturbed intracoronary hemodynamics in myocardial bridging: early normalization by intracoronary stent placement. Circulation.

[8] Downey HF, Crystal GJ, Bashour FA (1983). Asynchronous transmural perfusion during coronary reactive hyperaemia. Cardiovasc Res.

[9] Gould KL, Johnson NP (2015). Myocardial bridges: lessons in clinical coronary pathophysiology. JACC Cardiovasc Imaging.

[10] Hung OY, Brown AJ, Ahn SG, Veneziani A, Giddens DP, Samady H (2015). Association of wall shear stress with coronary plaque progression and transformation. Interv Cardiol Clin.

[11] Corban MT, Hung OY, Eshtehardi P (2014). Myocardial bridging: contemporary understanding of pathophysiology with implications for diagnostic and therapeutic strategies. J Am Coll Cardiol.

[12] Wu S, Liu W, Zhou Y (2016). Spontaneous coronary artery dissection in the presence of myocardial bridge causing myocardial infarction: an insight into mechanism. Int J Cardiol.

[13] Lovell MJ, Knight CJ (2003). Invasive assessment of myocardial bridges. Heart.

[14] Duymun S, Misodi E (2020). Myocardial bridging: a case presentation of atypical chest pain syndrome in a young woman. Am J Case Rep.

[15] Hazirolan T, Canyigit M, Karcaaltincaba M, Dagoglu MG, Akata D, Aytemir K, Besim A (2007). Myocardial bridging on MDCT. AJR Am J Roentgenol.

[16] Schwarz ER, Gupta R, Haager PK, vom Dahl J, Klues HG, Minartz J, Uretsky BF (2009). Myocardial bridging in absence of coronary artery disease: proposal of a new classification based on clinical-angiographic data and long-term follow-up. Cardiology.

[17] Kikuchi S, Okada K, Hibi K (2018). Myocardial infarction caused by accelerated plaque formation related to myocardial bridge in a young man. Can J Cardiol.

[18] Ide T, Ohtani K, Higo T, Tanaka M, Kawasaki Y, Tsutsui H (2019). Ivabradine for the treatment of cardiovascular diseases. Circ J.

[19] Prendergast BD, Kerr F, Starkey IR (2000). Normalisation of abnormal coronary fractional flow reserve associated with myocardial bridging using an intracoronary stent. Heart.

